# Analysis of the impact of different schemes of preparation to trabeculectomy on the healing markers on the Tenon fibroblasts cultures

**DOI:** 10.1038/s41598-023-43246-z

**Published:** 2023-09-28

**Authors:** Joanna Piłat, Agata Przekora, Dominika Wróbel-Dudzińska, Paulina Kazimierczak, Tomasz Żarnowski, Ewa Kosior-Jarecka

**Affiliations:** 1 Department of Diagnostics and Microsurgery of Glaucoma, Medical University of Lublin, Ul. Chmielna 1, 20-079 Lublin, Poland; 2 Independent Unit of Tissue Engineering and Regenerative Medicine, Medical University of Lublin, Lublin, Poland

**Keywords:** Diseases, Medical research

## Abstract

The aim of the study was to assess the influence of different regimes of patient’s preparation before trabeculectomy on the markers of healing process in Tenon’s fibroblast cultures.The studied group consisted of 66 patients with open angle glaucoma undergoing primary trabeculectomy. The patients were divided into 5 groups with different regimes of preparation before the surgery based on application or withdrawal of topical antiglaucoma medications and steroids (G1—patients using antiglaucoma drops until the day of the surgery; G2—patients using antiglaucoma drops until the day of the surgery and additionally dexamethasone for 4 weeks before surgery; G3—patients who stopped using antiglaucoma drops 4 weeks before the surgery and introduced dexamethasone for 4 weeks before surgery; G4—patients who stopped using antiglaucoma drops 4 weeks before the surgery; G5—control group, patients with newly diagnosed glaucoma in whom trabeculectomy was the first treatment option without medical treatment). During trabeculectomy the samples of Tenon’s capsule were obtained. Tenon fibroblasts were isolated directly from the explants to test their proliferation ability and the level of released healing markers. Following factors typical of healing process were evaluated using commercially available ELISA kits: IL 1-β, IL-6, IL-8, VEGF-A, TGF-β1 and MMP-9. Concentrations of IL-1β, IL-6 and TGF-β1 were significantly higher in the group obtaining antiglaucoma drops. Additionally, in this group the fibroblasts revealed the highest proliferation potential, indicating the active healing process. The levels of IL-8, VEGF-A and MMP-9 were similar between the groups. Our study shows that for the best conjunctival anti-inflammatory control, the most influential factor is the withdrawal of antiglaucoma medications.

## Introduction

Glaucoma is a group of multifactorial eye diseases that result in progressive irreversible damage of the optic nerve and cause vision loss and is considered the second leading cause of blindness in the world. The main element in the pathophysiology of glaucoma is the loss of retinal ganglion cells and their axons, which leads to changes in the morphology of the optic disc and consequently to visual field defects. The main risk factor for glaucoma development and progression is elevated intraocular pressure (IOP). All effective strategies in the treatment of glaucoma are based on the IOP decrease which could be obtained by medical or surgical management.

Trabeculectomy is a surgical procedure in glaucoma which evolved since its introduction in 1968^1–3^ and became the golden standard surgical option for glaucoma, as evidence from clinical trials such as the Collaborative Initial Glaucoma Treatment Study and the Advanced Glaucoma Intervention Study showed that trabeculectomy could sufficiently minimise progression of glaucomatous neuropathy. During the surgery the fistula between anterior chamber and subconjunctival space is created to promote a new pathway for aqueous humour outflow^3^. The meticulous postoperative period is fundamental to its success and can be more challenging than the procedure itself since it is associated with many complications. The most frequent cause of late failure of trabeculectomy is malfunction of the filtering bleb associated with IOP increase and caused by excessive subconjunctival scarring^4^.

The surgical injury caused by trabeculectomy induces a cascade of pathophysiological reactions causing tissue fibrosis in the bleb region and therefore minimising the aqueous humour outflow. The exposure of fibroblasts to proinflammatory factors promotes phenotypic changes leading to increases in proliferation and migration, and tissue remodelling. Inflammation occurs in an early phase of wound healing and is attributable to immune cell activation of neutrophils and macrophages which infiltrate into a wound and produce proinflammatory cytokines and chemokine. Various proinflammatory cytokines released by infiltrating inflammatory cells induce mesenchymal cell and fibroblast activation, migration and finally transformation into myofibroblasts. Myofibroblasts, transformed and activated by proinflammatory cytokines, produce contractile proteins which cause extracellular matrix (ECM) reorganisation and loss of tissue functionality^5^.

The controlling of the healing processes after trabeculectomy is the key factor to keep persistence of outflow and bleb function. This goal is realised at the different stages of the surgery. First, preoperatively, the minimising of the conjunctival inflammation connected with the prolonged medical treatment is introduced. During the surgery antiproliferative agents (5-fluorouracil or mitomycin C) are applied in different concentrations and time, aimed at controlling postoperative healing. Although they are potent healing inhibitors, their usage is limited by the risk of complications, some severe leading to blindness, such as persistent leakage of the filtering bleb, clinical hypotony and bleb-related endophthalmitis. After the surgery prolonged topical steroid application and intensive care of the bleb with massage and removal of releasable sutures^4,6^.

There is no clear scheme of preparation of the patients before trabeculectomy. Usually, in the preoperative period, according to the clinical presentation of patients, medical therapy is remodulated with the attempt to simplify the therapy regimen.

However, there is only scant of evidence for such regimen. An “inflammatory activated conjunctiva” may reduce the likelihood of trabeculectomy success as the prolonged application of antiglaucoma medical treatment causes and promotes conjunctival fibrosis^7–9^. Local steroid application before trabeculectomy would diminish the fibroblastic activity with inhibition of wound healing. There is some evidence to show that a 1-month course of fluorometholone^10^ will improve the state of the conjunctiva preoperatively. It is generally assumed that such preoperative preparation should improve the likelihood of trabeculectomy success, though the evidence base regarding time and manner of preoperative medical treatment before trabeculectomy is still weak.

The aim of the study was to assess the influence of different regimes of patient’s preparation before trabeculectomy on the markers of the healing process.

## Materials and methods

### Studied groups

The studied group consisted of 66 patients undergoing trabeculectomy at the Department of Diagnostics and Microsurgery of Glaucoma, Medical University of Lublin, Poland between 2018 and 2020. All participants wrote informed consent before enrolment. The study adhered to the tenets of the Declaration of Helsinki, and the study design was approved by the Bioethics Committee of the Medical University of Lublin (0254/138/2016).

At the inclusion the following parameters were assessed: BCVA, using Snellen charts with decimal scale; slit lamp biomicroscopy with the evaluation of the anterior segment of the eye; gonioscopy, using Zeiss four-mirror gonioscope; as well as the stereoscopic fundus examination of the eye with a detailed assessment of the optic disc morphology. IOP was measured by Goldman applanation tonometry. Additionally visual field evaluation was performed with Humphrey Field Analyzer 745i (Carl Zeiss). The detailed general and ophthalmic histories were obtained. Only the patients fulfilling all inclusion criteria but none of exclusion criteria were included.

Inclusion criteria were as follows:

1. Primary open angle glaucoma or pseudoexfoliative glaucoma
  The open angle glaucoma was defined according to the presence of glaucomatous neuroretinal rim loss, glaucomatous VF damage in at least three valuable perimetric tests and an open angle in gonioscopy. The diagnosis of pseudoexfoliation syndrome was based on the presence of dandruff-like exfoliative material on the anterior lens capsule in the central disc and peripheral band (double concentric ring) pattern and/or in the anterior segment of the eye. The criteria of glaucoma diagnosis in PEX syndrome were the same as mentioned above.
2. Application of the same scheme of antiglaucoma drops (latanoprost + dorzolamide + timolol) for at least 6 months before enrolment. The same scheme antiglaucoma therapy was applied to avoid bias in conjunctival status caused by drops with different mechanisms of action.
3. Planned trabeculectomy. Trabeculectomy was performed in all patients if the target pressure was not obtained by maximal medical therapy. Group 5 include untreated patients with newly diagnosed advanced glaucoma with high IOP in whom according to the results of the AGIS^11^, primary trabeculectomy would better stabilise the disease.

Exclusion criteria were as follows: angle closure glaucoma, neovascular glaucoma, uveitic glaucoma, silicone oil glaucoma, previous antiglaucoma surgery or any surgery involving conjunctiva or sclera, previous argon laser trabeculoplasty, coexisting connective tissue diseases, haematologic or immunologic disorders, diabetes mellitus, general or local immunomodulating treatment.

Patients included to the study were randomly assigned to one of the groups and asked to use the specified preparation scheme before the planned trabeculectomy. The exception was group 5 which involved patients previously untreated. Patients were asked to apply different scheme of preparation 4 weeks before planned trabeculectomy:

- Group 1—patients using antiglaucoma drops until the day of the surgery.
- Group 2—patients using antiglaucoma drops until the day of the surgery and additionally dexamethasone (Dexamethasone phosphate 1 mg/1 ml, without preservatives) 4 times a day for 4 weeks before surgery.
- Group 3—patients who stopped using antiglaucoma drops 4 weeks before the surgery and introduced dexamethasone (Dexamethasone phosphate 1 mg/1 ml, without preservatives) 4 times a day for 4 weeks before surgery. Patients obtained oral acetazolamide 2 × 250 mg to control IOP.
- Group 4—patients who stopped using antiglaucoma drops 4 weeks before the surgery. Patients obtained oral acetazolamide 2 × 250 mg to control IOP.
- Group 5—control group, patients with newly diagnosed glaucoma in whom trabeculectomy was the first treatment option without medical treatment.

Initially, 66 patients with POAG or PEXG were enrolled to the study and after preparation according to the scheme have performed trabeculectomy. During the surgery the samples of the Tenon's layer were obtained as described later. In 13 patients (19.69%) enough of the cells during tissue culture was not obtained, 5 patients (7.58%) were excluded because of the new circumstances fulfilling exclusion criteria. In 11 cases (16.67%) cultured cells died during defrosting. Final analysis was performed in 37 patients (56.06%).

Demographic characteristics of studied groups are put in Table 1.

**Table 1:** Demographic characteristics of the studied groups.

Group	1	2	3	4	5	p level
Number of patients	6	8	8	7	8	
Age	73.5 ± 6.7	71.2 ± 8.9	65.5 ± 9.6	66.3 ± 14.3	57.5 ± 3.2	*p* = 0.06
Gender	F1/M5	F5/M3	F3/M5	F4/M3	F1/M7	*p* < 0.05
Type of glaucoma POAG/PEXG	3/3	6/2	5/3	7/0	6/0	*p* < 0.05
BCVA	0.64 ± 0.36	0.75 ± 0.28	0.79 ± 0.32	0.79 ± 0.32	0.73 ± 0.33	*p* = 0.42
IOP at qualification to trabeculectomy [mmHg]	22 ± 7.3	25 ± 7.5	27 ± 5.9	22 ± 6.0	33 ± 11.6	*p* = 0.67
MD [dB]	− 20.00 ± 4.85	− 18.29 ± 9.20	− 15.98 ± 11.06	− 12.47 ± 8.60	− 18.78 ± 9.24	*p* = 0.65

### Material

On the day of the surgery the patient obtained 1 drop of 2% of Pilocarpine and Fluorochinolone (Levomer, AdamedPharma). Peribulbar anaesthesia was performed with injection of 5 ml of standard prepared anaesthetic mixture of lignocaine, bupivacaine, adrenaline and hyaluronidase. Before the surgery the drop of 5% povidone-iodine solution was installed. At the beginning of the surgery, transcorneal fixating suture was placed to visualise the upper quadrant of the bulb. The small conjunctival incision was performed near the limbus and conjunctiva and Tenon’s layer were bluntly dissected posteriorly. At this stage, the Tenon’s layer was visualised, and the small fragment (approx.1 × 2 mm) was cut with care not to take conjunctiva. The obtained material was placed in a 1.5 ml sterile Eppendorf tube fulfilled with the solution of 0.5 ml phosphate buffered saline (PBS, Sigma-Aldrich Chemicals) with 300 U/ml penicillin, 300 μg/ml streptomycin and 0,75 μg/ml amphotericin B (Sigma-Aldrich Chemicals). The surgery was further performed typically as limbus-based procedure with mitomycin C application and removable sutures. The obtained material was immediately delivered to the clean room for isolation of the cells.

### Methods

#### Isolation and culture of Tenon fibroblasts

Isolation and culture of Tenon fibroblasts was performed according to the previously developed and described procedure^1^. Briefly, at the beginning explant of Tenon capsule was twice washed with PBS solution and divided with the sterile blade into 2 or more pieces. The explant samples were then transferred to the wells of 12-multiwell plate and cultured in EMEM medium supplemented with: 5% fetal bovine serum (FBS, EU Professional grade, Pan-Biotech), 5 μg/ml recombinant human (rh) insulin, 5 ng/ml rh basic fibroblast growth factor (rh FGF b), 50 μg/ml ascorbic acid (components of Fibroblast Growth Kit, ATCC—LGC Standards), 7 mM L-glutamine (Sigma-Aldrich Chemicals), 100 U/ml penicilin, 100 μg/ml streptomycin, and 0.25 μg/ml amphotericin B. The culture conditions were as follows: 37 °C in a humidified atmosphere of 5% CO_2_ and 95% air. Half of the culture medium was changed every 3 days to enhance the cell growth. After formation of monolayer, the fibroblasts were detached by trypsinization and transferred to a 25 cm^2^ T-flask. The successful isolation of Tenon fibroblasts was confirmed by immunofluorescence staining of vimentin filaments as described earlier^12^. The cells were cryopreservated at third passage and stored in a liquid nitrogen until use. Before the planned tests the cells isolated from tissue explants of all patients were thawed in a water bath at 37 °C, and their viability was checked with trypan blue. Cells isolated from 11 tissue samples (16, 67%) did not retain sufficient viability to use them in the experiments. After thawing, the cells were cultured in a basal EMEM medium with 10% FBS and antibiotics (100 U/ml penicillin 100 μg/ml streptomycin) without other supplements used for isolation. The isolated fibroblasts were subjected to proliferation assay. Moreover, the level of healing markers and pro-inflammatory cytokines was assessed in the cell culture supernatants.

#### Cell proliferation assay

To evaluate the fibroblasts’ ability to proliferate, the cells after thawing were seeded at low density of 6 × 10^4^ cells/well into 12-multiwell plate. The cells were incubated for 2 days in 10% EMEM. The exact number of cells was calculated on seeding day and after 2-day culture by means of commercially available proliferation test—WST-8 (Cell Counting Kit-8, Sigma-Aldrich Chemicals), which was performed according to the manufacturer's instruction. Doubling Time (time in hours required to double cell population) of the Tenon fibroblasts was calculated using Doubling Time Computing software.

#### Evaluation of healing markers and pro-inflammatory cytokines

After thawing the part of fibroblasts suspension was seeded at density of 6 × 10^4^ cells/well into 12-multiwell plates and incubated in 10% EMEM in 37 °C for 24 h, then the supernatants were collected. The level of healing markers and pro-inflammatory cytokines was measured in the cell culture supernatants using commercially available ELISA kits. The following factors were evaluated: interleukin-1β (Interleukin-1 beta Human ELISA, BioVendor), interleukin-6 (Interleukin-6 beta Human ELISA, BioVendor), interleukin-8 (Interleukin-8 Human ELISA, BioVendor), Human vascular endothelial growth factor-A (Human VEGF-A ELISA Kit, Diaclone), Human transforming growth factor-β1 (Human TGF-beta1 ELISA Kit, Diaclone), and matrix metalloproteinase-9 (Human MMP-9 ELISA, BioVendor). All tests were performed according to the manufacturer's instructions. In order to obtain reliable data and exclude the effect of different cell density on the level of healing markers and pro-inflammatory cytokines, the ELISA results were normalized per 1 mg of cellular proteins. Total cellular proteins were assessed in cell lysates by Bradford colorimetric method using commercially available kit (Pierce™ BCA Protein Assay Kit, ThermoFisher Scientific). The test was performed according to manufacturer’s instructions. To obtain cell lysates, culture medium was discarded and replaced with PBS containing protease inhibitors (Sigma Aldrich-Chemicals). Cell lysis was performed by 3 freeze–thaw cycles and sonification with ultrasounds.

### Statistical analysis

The biochemical markers for each patient were evaluated by ELISA in 2 repetitions. The mean value was calculated and used for further analysis.

The results were statistically analysed with computer programs IBM SPSS Statistics v. 26 and STATISTICA 13 (StatSoft Polska) with ANOVA test for comparisons between the groups and Spearman correlation tests to look for the correlations. The statistically significant results were when p level was lower than 0.05.

## Results

### Proliferation potential

#### Fibroblast doubling time

**Figure 1:**
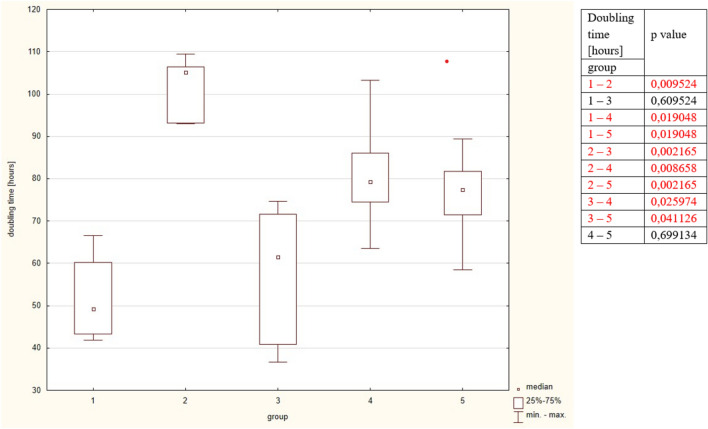
Doubling time in the studied group. The table shows the results of Mann–Whitney comparison between the groups, the red colour shows statistical significance.

The most actively dividing fibroblasts were observed in group 1, the statistical difference was observed in comparison to groups 2, 4 and 5 (test U Mann–Whitney. In group 2 fibroblast DT was the longest and the significant comparison was observed to all groups. In group 3 fibroblast DT was statistically shorter compared to groups 2, 4 and 5. The results did not differ between groups 4 and 5. The detailed results are put in Table 2 and Fig. 1.

**Table 2:** Concentrations of the studied healing markers in the studied groups.

Group	1	2	3	4	5	*p* level
Il 1-beta [pg/mg]	1.38 ± 0.38	1.08 ± 0.20	0.80 ± 0.016	0.79 ± 0.08	0.78 ± 0.10	*P* = 0.0053*
Il-6 [pg/mg]	902.75 ± 84.16	747.14 ± 96.71	591.45 ± 50.25	659.09 ± 64.11	592.11 ± 73.76	*P* = 0.0014*
Doubling time [hours]	51.8 ± 11.2	102.1 ± 7.2	57.8 ± 16.1	81.0 ± 13.3	75.9 ± 10.5	*P* = 0.0005*
Il-8 [pg/mg]	923.32 ± 267.41	692,46 ± 98.99	646.94 ± 155.57	662.52 ± 102.45	569.21 ± 149.75	*P* = 0.2074
VEGF [pg/mg]	1482.75 ± 843.77	955.92 ± 522.49	1277.52 ± 882.85	1420.15 ± 795.36	963.40 ± 662.53	*P* = 0.4261
TGF-β [pg/mg]	462.10 ± 126.40	381.46 ± 59.45	265.15 ± 44.85	298.68 ± 40.24	261.04 ± 33.43	*P* = 0.0016*
MMP-9 [pg/mg]	0.14 ± 0.03	0.66	0.75 ± 0.69	–	2.66 ± 3.61	–

*Statistically significant.

### Markers of inflammation

#### Interleukin-1 beta

**Figure 2:**
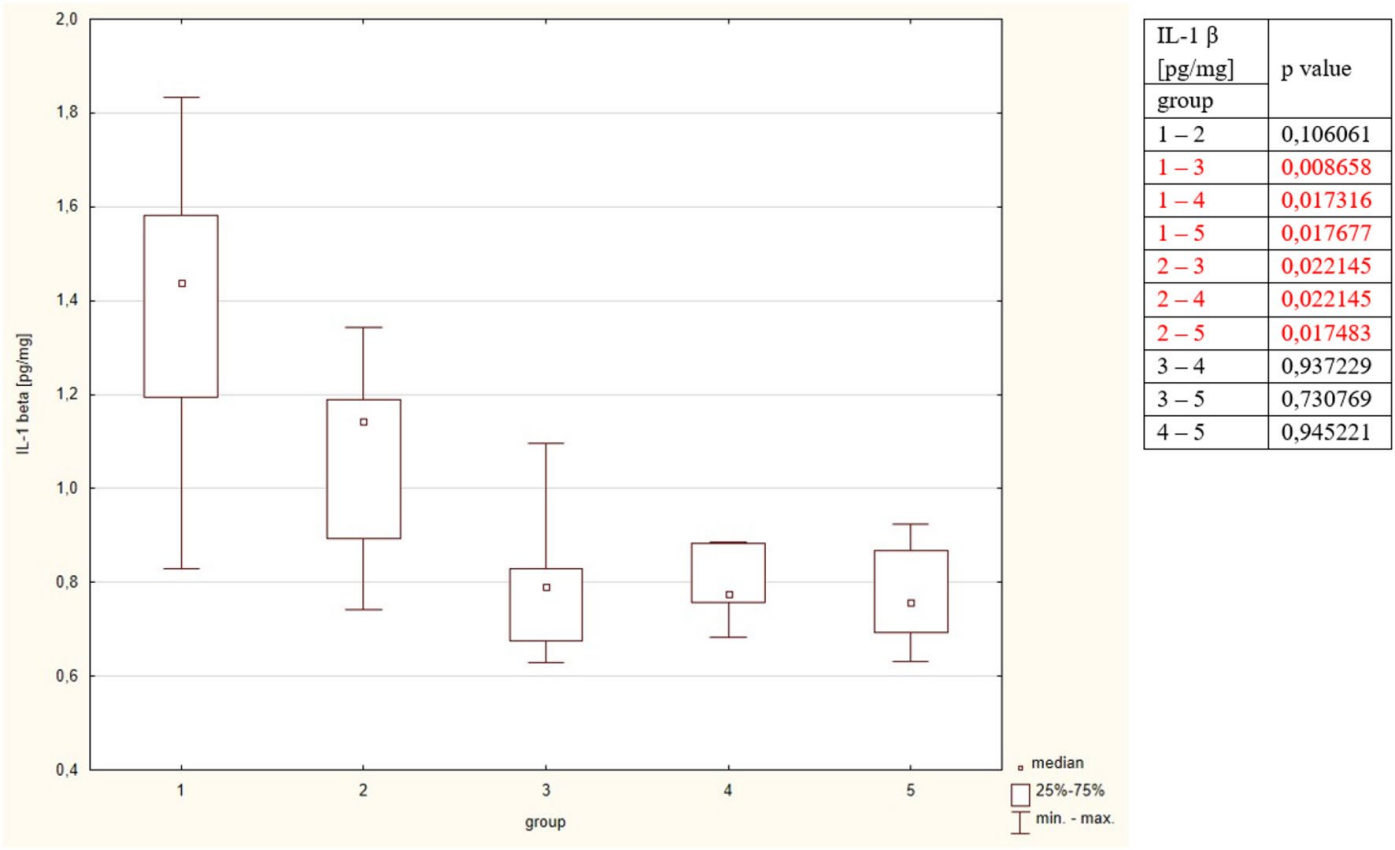
Concentrations of Il-1 beta in the studied group. The table shows the results of Mann–Whitney comparison between the groups, the red colour shows statistical significance.

Concentration of IL-1β was significantly higher in group 1 and compared to groups 3, 4, 5. The results did not differ between groups 1 and 2, and groups 3, 4, 5. The detailed results are put in Table 2 and Fig. 2.

### Interleukin-6

**Figure 3:**
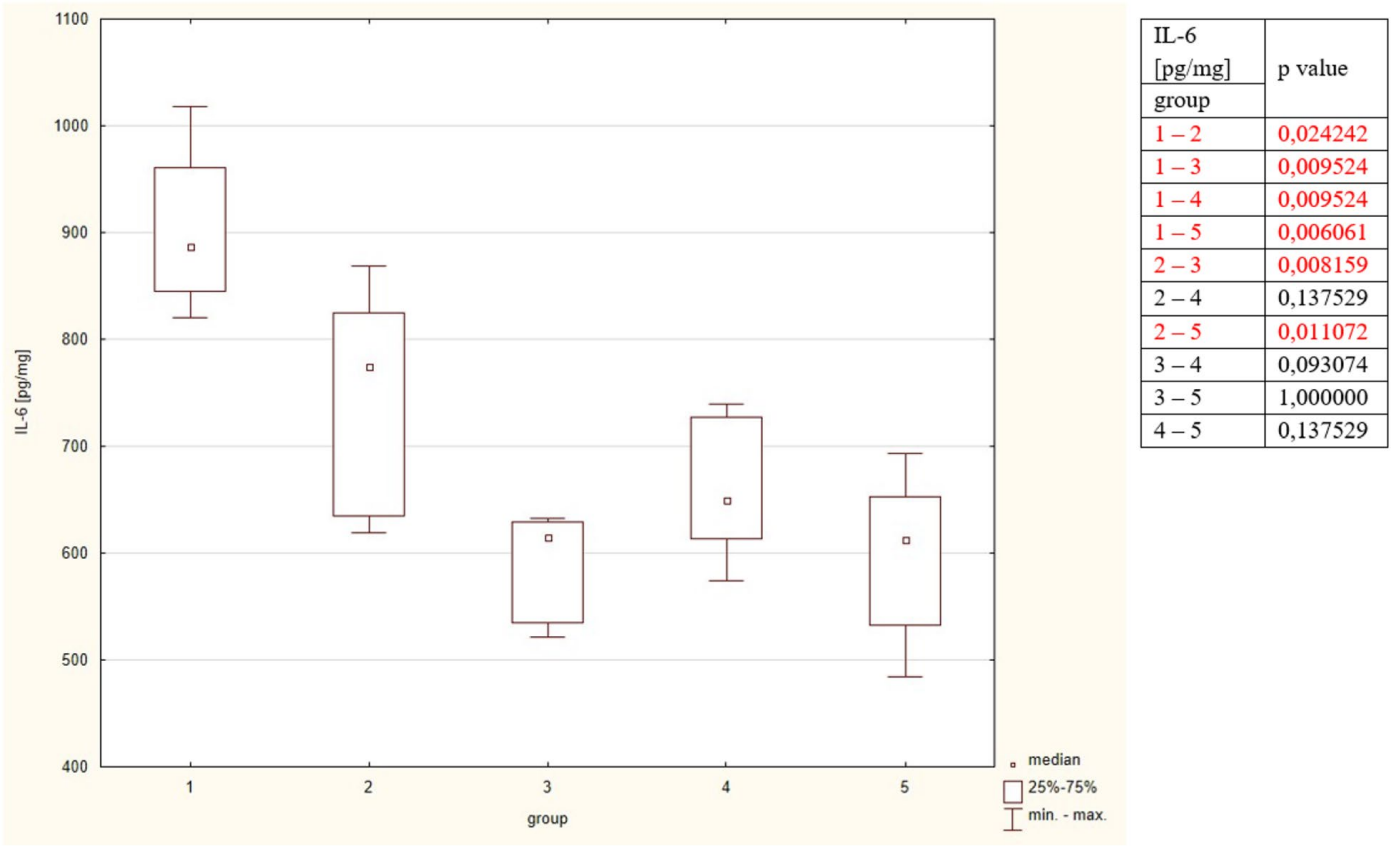
Concentrations of Il-6 in the studied group. The table shows the results of Mann–Whitney comparison between the groups, the red colour shows statistical significance.

The Il-6 concentration was statistically higher in group 1 compared to all remaining groups. In group 2 Il-6 concentrations were significantly compared to group 3 and 5. Groups 3, 4, 5 did not statistically differ regarding Il-6 concentration. The detailed results are put in Table 2 and Fig. 3.

### Markers of angiogenesis

#### Interleukin-8

**Figure 4:**
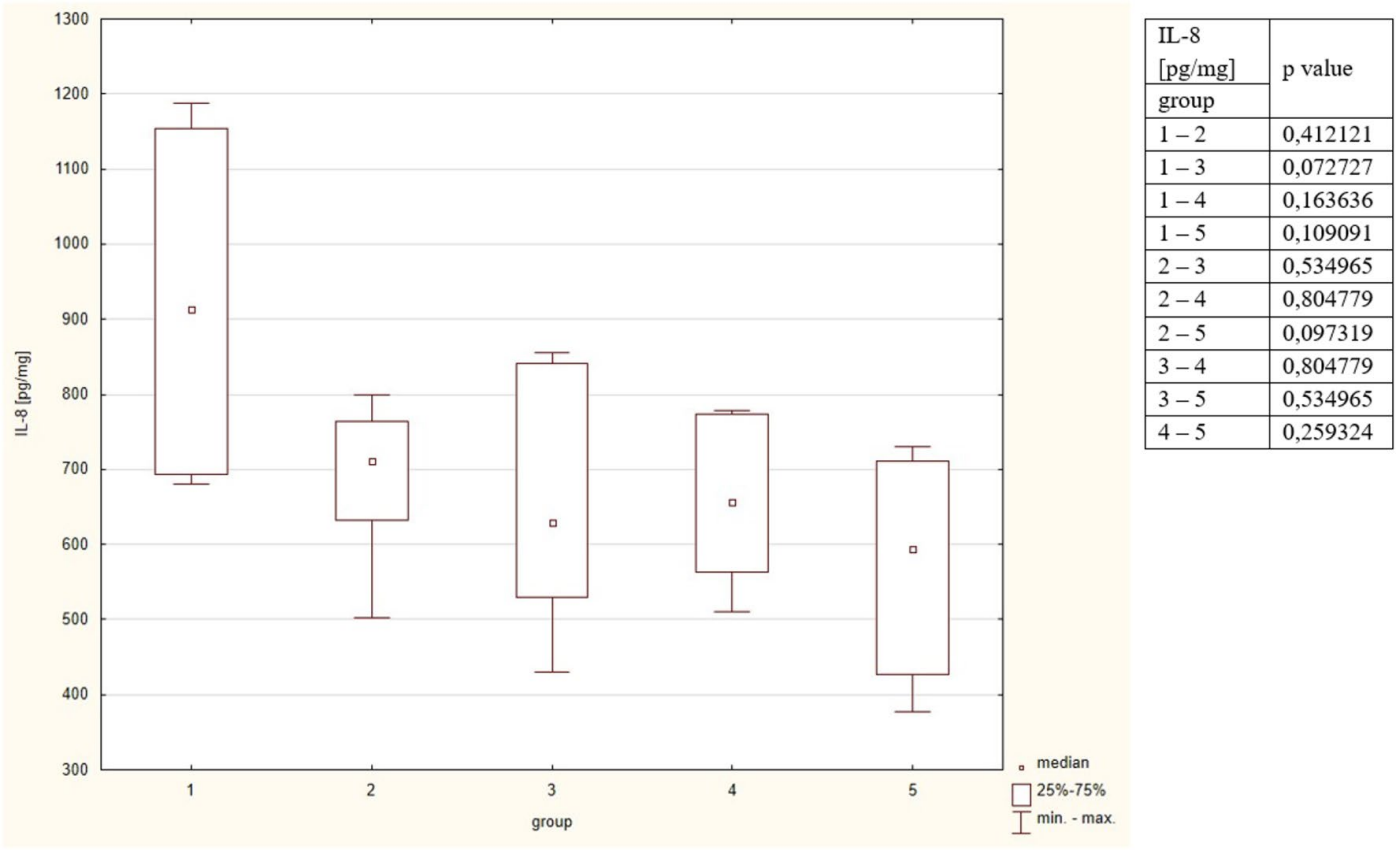
Concentrations of Il-8 in the studied group. The table shows the results of Mann–Whitney comparison between the groups, the red colour shows statistical significance.

The highest Il-8 concentration was observed in group 1, however, the difference was not significant compared to all groups. The detailed results are put in Table 2 and Fig. 4.

### VEGF-A

**Figure 5:**
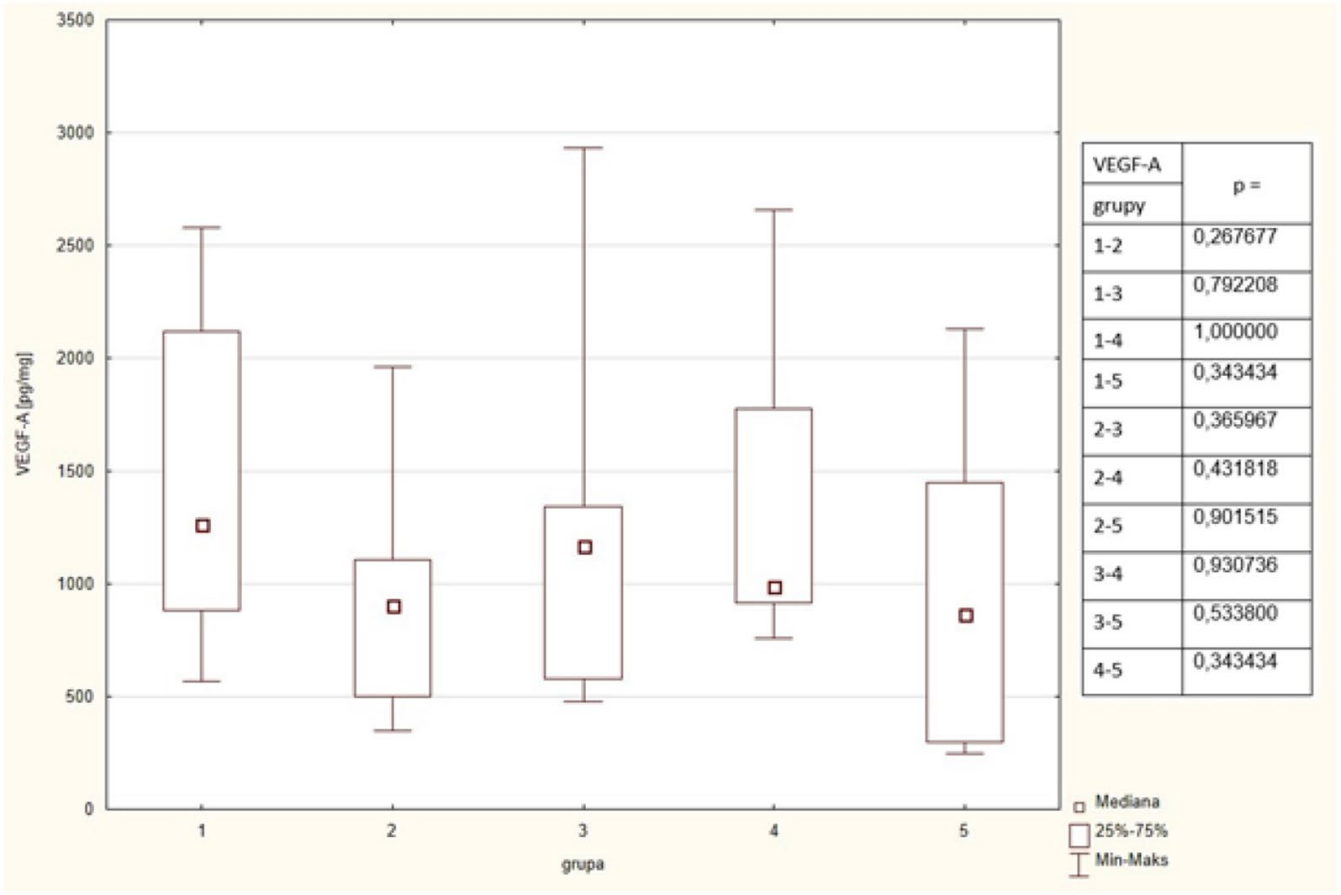
Concentrations of VEGF-A in the studied group. The table shows the results of Mann–Whitney comparison between the groups, the red colour shows statistical significance.

Mean VEGF-A concentrations were similar between the groups. The detailed results are put in Table 2 and Fig. 5. Positive correlation (rho = 0.5140) was observed between concentrations of Il-8 and VEGF.

### Markers of maturation and remodelling

#### Transforming Growth Factor-beta 1

**Figure 6:**
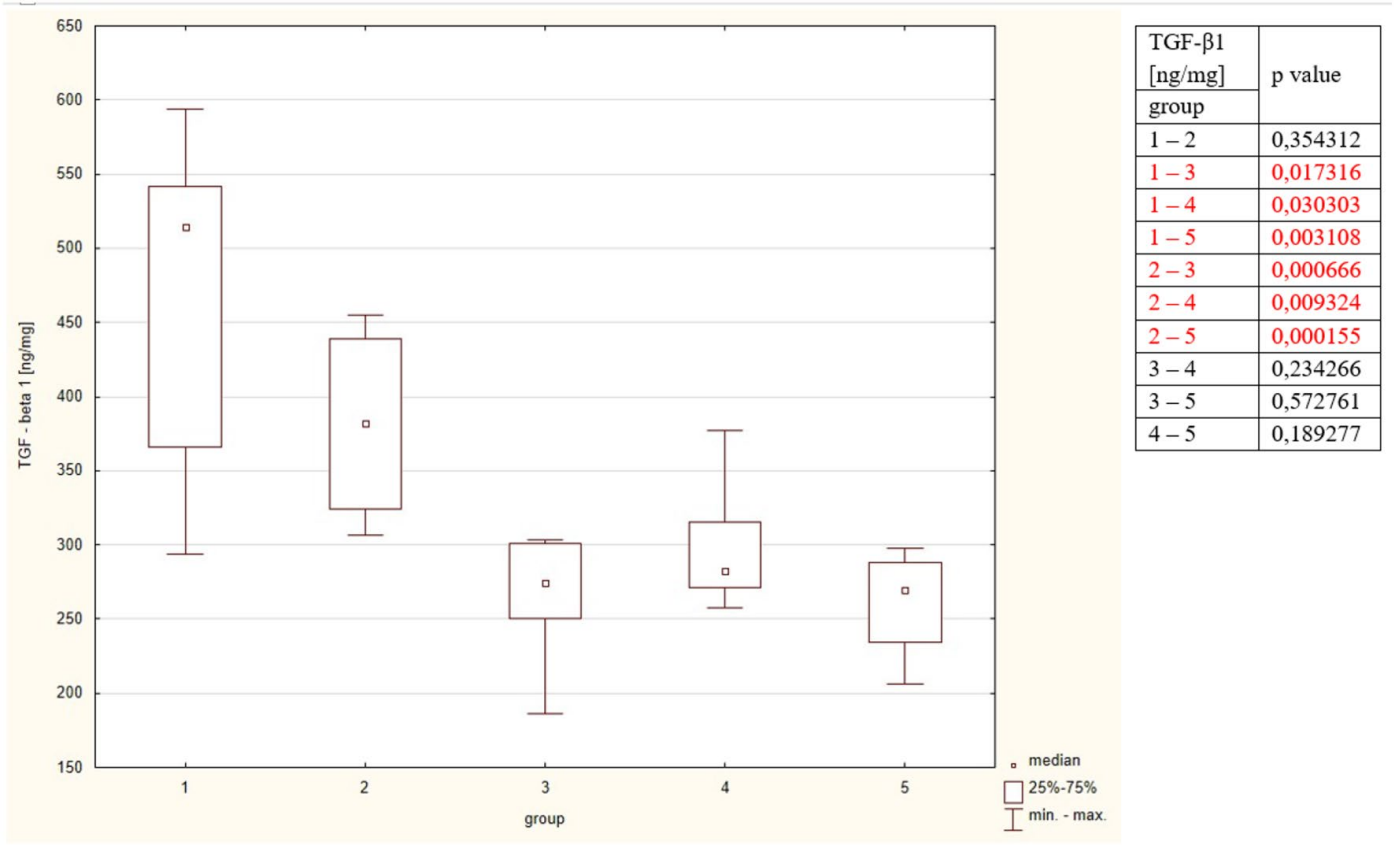
Concentrations of TGF-β1 in the studied group. The table shows the results of Mann–Whitney comparison between the groups, the red colour shows statistical significance**.**

Concentration of TGF-β1 was significantly higher in group 1 and compared to groups 3, 4, 5. The concentrations did not significantly differ between groups 1 and 2; and between groups 3, 4 and 5. The detailed results are put in Table 2 and Fig. 6.

### Matrix metalloproteinase-9

In all studied groups the detectable MMP-9 values were obtained from 8 patients (3 from group 1, 1 from group 2, 2 in group 3, none in group 4 and in group 5-2 patients). The data were not sufficient for the statistical analysis. The results are put in Table 2

## Discussion

The final long-term success of trabeculectomy is related to sufficient management of healing processes. The rate of clinical failures is higher in patients chronically treated with antiglaucoma drops^13^, because of induced prolonged conjunctival inflammation as the response for toxic and allergic actions of both active substances and preservatives^14–16^. In patients before trabeculectomy to diminish chronic inflammation of the ocular surface preoperatively, most of the surgeons withdraw antiglaucoma drops and introduce local steroid application. However, the detailed scheme of preparation schedule is rather intuitive, there are no clear indications. In this study we aimed to check the influence of steroids and antiglaucoma medications on inflammatory parameters which may be related to the subsequent wound healing. Two main factors were changed in our preoperative schemes: antiglaucoma drops which were long term used as maximal medical therapy and withdrawn in some groups 4 weeks before planned surgery, and local dexamethasone application introduced in some groups of patients. The time of the preoperative therapy was related to the fact that for most antiglaucoma drops wash-out time lasts a month^17^. This time seems to be sufficient, the studied parameters were statistically similar between the group with stopped drops and the untreated control group. To facilitate the patients fulfilling the preoperative schedule, the steroids were introduced in a similar manner. The previous studies showed that steroid installation for 3–4 weeks before trabeculectomy decreases the failure rate in long term observation^8,18^. However, prolonged steroid treatment in some glaucoma patients may cause IOP increase. In this study, we used dexamethasone as a potent steroid which is used in our typical practice in trabeculectomy patients and which at the time of study design was the only preservative-free steroid medicament in drops commercially available in Poland. Although, some previous research^15,16^ used fluorometholone as the one with the smaller potential to cause IOP spikes.

IL1-β is produced by macrophages, fibroblasts, and vascular endothelial cells at the early stage of injury^19^ which enhances the inflammatory reaction. Increased IL-1β level was observed in tear film, conjunctiva, and aqueous humour of glaucoma patients but also in patients with dry eye syndrome and Meibomian gland dysfunction, frequent in patients on antiglaucoma medical treatment^20,21^. In our study, increased IL-1β level in Tenon’s fibroblasts culture was observed in patients constantly installing antiglaucoma drops. The withdrawal of antiglaucoma drops was the most important factor in decreasing IL-1β level; a 1-month antiglaucoma medication free declined the IL-1β level to the level observed in the naive patients. The introduction of corticosteroids was not a significant factor.

IL-1β-activated lymphocytes, macrophages, fibroblasts and vascular endothelial cells produce IL-6, whose concentration increases 12-16 h after the injury and further promotes inflammatory reactions^22^. Enhanced IL-6 expression was shown in conjunctiva of patients constantly using timolol^23,24^ and latanoprost^25^, which seemed preservatives independent^23^. Our results confirm these results, IL-6 level was the highest in the group of patients permanently applying antiglaucoma drops, application of steroids significantly augmented IL-6 level decrease. This drug combination likens IL-6 level to the one observed in untreated control. Dexamethasone directly reduces IL-6 expression but also inhibits its IL-1β induced production^26,27^.

In the wound healing after trabeculectomy, the early inflammatory phase initiates proliferation resulting in formation of loose granulation tissue consisting of fibroblasts, newly formed vessels, macrophage, and collagen fibres. Tenon’s capsule fibroblasts are the main cells involved in the tissue repair after surgery, their ability to migrate and proliferate reflects the healing potential. Sherwani et al.^28^ showed that prolonged medical antiglaucoma treatment increases the number of fibroblasts. In this study evaluating proliferative potential of fibroblasts, the fibroblasts obtained from the patients constantly using antiglaucoma drops were significantly more active. The most beneficial effect was observed in case of combined antiglaucoma and steroid therapy. It may reflect cumulative proapoptotic effects of preservatives from antiglaucoma drops and steroids, prevailing the beneficial effect of hypotensive medication withdrawal. The studies showed that for different antiglaucoma medications, the same apoptotic index was observed in case of the same number of BAK, no matter the active antiglaucoma compound^29,30^. Surprisingly, in a group of patients washed-out from glaucoma medications and using steroids, the proliferative potential of fibroblasts was similar to the group obtaining constant antiglaucoma drops, which was significantly higher to all remaining groups. The expected results in the steroid group would be a significant decrease in fibroblast proliferation due to diminishing influence on the proinflammatory cytokine production and cell cycle. However, the increased proliferative activation after dexamethasone treatment was observed also for other tissues^31,32^, the mechanisms remain elusive.

The other process crucial during the proliferative stage of healing is angiogenesis concurrent to dynamic fibroblast ingrowth. From the 4th day of injury, the most important proangiogenic factor in the granulation tissue is VEGF produced by fibroblasts and macrophages. It increases vascular permeability and has a promitotic influence on vascular endothelial cells. Patients applying antiglaucoma drops for prolonged periods of time show conjunctival redness resulting from chronic inflammation. However, there is only a scant amount of the research evaluating impact on antiglaucoma medications on VEGF status, the different classes of the drugs seem to have different influences^33^, similarly to the relation between dexamethasone and VEGF^34–36^. Our study also reflects these tendencies: the Tenon’s fibroblasts cultures showed high fluctuations not correlated to any applied schemes in preparation for trabeculectomy. On the other hand, it is probable that the main factor triggering angiogenesis is the surgical wound and no antiglaucoma drops nor steroid application in preoperative period influence proangiogenic factors.

IL-8 also promotes angiogenesis stimulating mitosis and vascular endothelial cells migration creating scaffolding for newly formed vessels^37^. High IL-8 level was described in patients with dry eye syndrome, allergic conjunctivitis and Moeibomian gland dysfunction. In glaucoma patients increased IL-8 concentrations were shown in aqueous humour, tears and conjunctiva^19,38^. Additionally, antiglaucoma medical therapy increases IL-8 expression^14,23^. In this study, IL-8 level was not significantly higher in patients using persistent antiglaucoma medications, which was slightly suppressed by dexamethasone addition. The suppressing action of dexamethasone on IL-8 synthesis was previously described^39,40^ on fibroblasts obtained from inflammatory orbit.

One of the crucial factors in every stage of wound healing is TGF-β1released in the early phase, which has a chemotactic influence on inflammatory cells, enhances fibroblast proliferation and boosts VEGF production. It is also engaged in late healing stages initiating remodelling and maturation of the scar by MMP synthesis and collagen production^37^. Its expression in the eye is enhanced in case of ocular surface inflammation observed in dry eye syndrome and Moeibomian gland dysfunction, frequently coexisting with prolonged medical treatment of glaucoma. Moreover, TGF-β1 induced fibroblast transformation into active myofibroblasts is the mechanism of trabecular meshwork, astrocytes, and lamina cribrosa transformation during glaucoma^41^. The small studies regarding the influence of antiglaucoma drops on TGF-β1 level showed its increased level after prostaglandin analogue installation especially when combined with preservatives^42,43^. Our study showed that in a group of patients applying antiglaucoma medications until the time of trabeculectomy TGF-β1 level was the highest, which was only slightly decreased by adding dexamethasone. However, suppressing action of steroids on TGF-β1 was described previously^44–47^. Our results regarding TGF-β1 strongly encourage antiglaucoma drops withdrawal before penetrating surgery.

Maturation stage during the wound healing is connected to active myofibroblast shrinkage and collagen production, which are mediated by TGF-β1, and extracellular matrix remodelling moderated by MMPs. MMP-9 cleavages collagen type III increases myofibroblast shrinkage^48^. Elevated MMP-9 level was shown in excessive scarring^37^. There are limited studies concerning the influence of antiglaucoma treatment on MMP-9 level, latanoprost was described to increase its concentration^42,43,49^. In this study, MMP-9 was rarely detected no matter the applied preparation regime.

To sum up, prolonged application of antiglaucoma drops before trabeculectomy in preoperative period results in high concentrations of the factors promoting healings in Tenon’s capsule, which may be related to late bleb failure and lack of postoperative success. The introduction of dexamethasone enabled for their decrease and in the studied groups the clinical success in 30% IOP decrease was highest in group 3 and the lowest in group 1. However, our patients using preoperative steroids tended to have more frequently early wound leakage and hypotony (unpublished data), which is concordant with other clinical studies showing that introduction of the steroids did not improve the results of trabeculectomy but increased the risk of complications^50,51^. On the other hand, our results did not focus on the influence of intraoperative applications of antimetabolites and postsurgical topical steroids as the branches for healing control.

Our study shows that for the best conjunctival anti-inflammatory control, the most influential factor is to withdraw the topical antiglaucoma medications, with IOP control with oral acetazolamide if needed.

## Data Availability

All the data are available on request from the corresponding author.
